# The value of the pragmatic-explanatory continuum indicator summary wheel in an ongoing study: the bullous pemphigoid steroids and tetracyclines study

**DOI:** 10.1186/1745-6215-13-50

**Published:** 2012-04-27

**Authors:** Daniel J Bratton, Andrew J Nunn, Fenella Wojnarowska, Gudula Kirtschig, Anna Sandell, Hywel C Williams

**Affiliations:** 1 MRC Clinical Trials Unit, London, UK; 2 Nuffield Department of Clinical Medicine, University of Oxford, Oxford, UK; 3 Department of Dermatology, Vrije Universiteit Medisch Centrum, Amsterdam, MB, 1007, Netherlands; 4 Centre of Evidence-Based Dermatology, Nottingham University Hospitals NHS Trust, Nottingham, UK

**Keywords:** BLISTER, Bullous pemphigoid, Explanatory, Pragmatic, PRECIS, Clinical trial

## Abstract

**Background:**

The Pragmatic-Explanatory Continuum Indicator Summary (PRECIS) tool is intended to be used in the design phase of trials to help investigative teams design trials in-line with their purpose. Our team applied this tool to an ongoing trial (BLISTER) to determine whether the initial suggestion among some team members that the trial could be described as largely pragmatic was the consensus.

**Methods:**

Each of the six members of the BLISTER trial team was sent a blank PRECIS wheel to independently complete. The results obtained were averaged and plotted on a single PRECIS wheel to illustrate the degree of pragmatism of the trial.

**Results:**

The trial team found that the design of the trial was closest to the pragmatic end of the pragmatic-explanatory continuum. The strongest consensus was found on the ‘flexibility of the comparison intervention’ and ‘practitioner adherence’ domains (SD = 13). The trial team appeared to disagree most on the ‘eligibility criteria’ (SD = 35) and ‘participant compliance’ (SD = 31) domains, although the large standard deviations were a result of a single outlier in the two domains.

**Conclusion:**

The PRECIS tool can be used to retrospectively determine the pragmatism of a trial provided enough expertise and information on the trial is available. Illustrating the design of a trial on the PRECIS wheel can help research users more easily identify studies of interest. We hope our recommendations for applying this useful tool will encourage others to consider using it when designing, conducting and reporting studies.

**Trial registration:**

Current Controlled Trials http://www.controlled-trials.com/ISRCTN13704604

## Background

### Pragmatic and explanatory trials

Clinical trials are sometimes described as being either pragmatic or explanatory. A pragmatic clinical trial is designed to test treatment effectiveness under real life conditions. To achieve this, pragmatic trials are designed to mirror current clinical practice by using active comparators and by permitting flexibility in the way interventions are used. Typically a wide range of participants with co-morbidities and compliance are included that more readily reflect clinical practice to increase the external validity or generalizability of results. By contrast, explanatory clinical trials are more restrictive and address treatment efficacy under ideal conditions. Explanatory trials typically recruit a narrow spectrum of patients who are likely to respond to therapy, trial treatments are likely to be standardised and closely controlled, and follow-up may be much more intensive than that undertaken in clinical practice. Explanatory trials typically answer the question ‘can this treatment work?’ whereas pragmatic trials are more geared towards answering the question ‘does this treatment work in clinical practice?’. This pragmatic-explanatory dichotomy is over-simplistic because many trials have features of both; hence the concept of a pragmatic-explanatory continuum exists [1,2].

### The pragmatic-explanatory continuum indicator summary wheel

**Figure 1:**
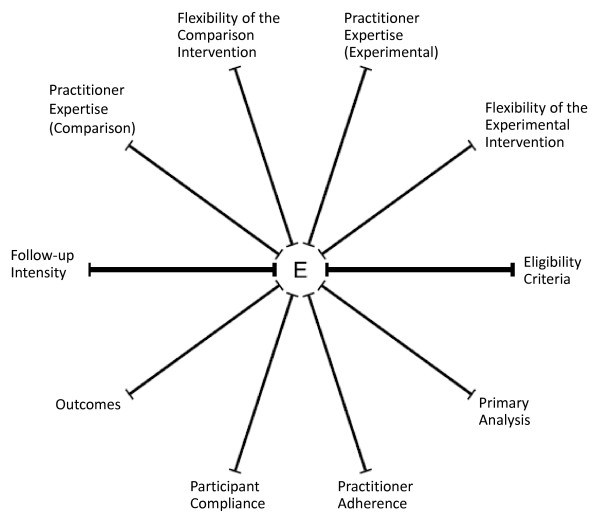
**The Pragmatic-Explanatory Continuum Indicator Summary (PRECIS) wheel **[2].

The Pragmatic-Explanatory Continuum Indicator Summary (PRECIS) wheel is a relatively new and useful tool for determining the position of a trial’s design in the pragmatic-explanatory continuum [2]. It is intended to be used in the design phase of trials to help determine whether the trial design fits with its intended purpose and perspective. The wheel assesses 10 key characteristics of a trial: eligibility criteria, flexibility of the experimental and comparison interventions, practitioner expertise of the experimental and comparison interventions, follow-up intensity, primary outcome, participant compliance, practitioner adherence and analysis of the primary outcome. A detailed description of each of these is given in the paper by Thorpe *et al*. [2]. The characteristics are represented by 10 spokes of a wheel (see Figure 1), each of which represents a pragmatic-explanatory continuum with the most pragmatic design marked on the rim and the most explanatory design at the centre. By mapping the features of a trial’s design onto each of these 10 domains, readers can gain an immediate visual sense of the extent to which a trial as a whole and, in particular, which domains are suggestive of a pragmatic or explanatory perspective.

Whilst the PRECIS tool seems to have good potential for practical use, little has been written on its application for those designing and conducting clinical trials. The PRECIS tool was recently used by a team designing a trial of a pain-coping skills training intervention for patients scheduled for knee arthroplasty [3]. In that study, the authors reported that the tool aided discussion on some trial design areas that may not have otherwise been discussed, and it helped the team reach a stronger consensus on the design of the trial. Another group used the PRECIS tool to estimate the degree to which randomised controlled trials included in two systematic reviews were predominantly pragmatic or explanatory [4].

In a study by Glasgow *et al*., nine reviewers each applied the PRECIS wheel to three weight loss studies with each PRECIS domain split into a five-point scoring system [5]. The authors then reported the average score in each domain and plotted these onto a single PRECIS wheel for each of the three trials. Categorising each domain into a limited number of points removes the continuous element of the PRECIS tool but if a sufficient number of points are used (for example, at least 10) then the loss of information should be minimal. Furthermore, such a scoring system may make the application of the PRECIS tool easier.

A study by Tosh *et al*. also used the five-point scoring system described above and applied it to mental health trials [6]. The scores on each wheel were summed onto a 0 to 50 scale, effectively reducing the multidimensional PRECIS tool to a single dimension. The authors interpreted a trial scoring 0 to 30 as explanatory and more than 35 as pragmatic, with the interim representing trials which ‘balance pragmatic and explanatory domains’ [6]. Such an interpretation does not acknowledge the existence of the pragmatic-explanatory continuum, meaning that two trials classified as pragmatic cannot be distinguished even if one may be considerably more pragmatic than the other.

In this descriptive study, we describe the use of the PRECIS tool in helping to resolve debate within the trial team on whether an ongoing clinical trial of two drug treatments in a rare blistering skin disease was more pragmatic or explanatory. We also provide practical suggestions on how best to use the PRECIS tool in such a context.

### The bullous pemphigoid steroids and tetracyclines study

The bullous pemphigoid steroids and tetracyclines study (BLISTER, ISRCTN13704604) is a randomised non-inferiority trial that seeks to determine whether doxycycline (200 mg/day) is non-inferior to prednisolone (0.5 mg/kg/day) in the treatment of bullous pemphigoid. Doxycycline is unlikely to be more effective than oral steroids for inducing remission and controlling blister formation in this autoimmune blistering disease, but it is likely to have some degree of efficacy and is probably much safer in the long-term than oral steroids [7,8]. The latter can, in an extreme scenario, lead to premature death in the predominantly elderly population that develop pemphigoid. Non-inferiority will be concluded in this trial if both the short-term control of pemphigoid in the doxycycline arm is not worse than that in the prednisolone arm by more than the pre-specified non-inferiority margin and if doxycycline is superior in terms of long-term safety.

Throughout the development of the BLISTER trial, members of the trial management group (TMG) debated whether BLISTER could be considered to be a pragmatic or explanatory trial. While some members considered that the trial was more towards the pragmatic end of the spectrum, others were less certain given the participant inclusion criteria and the standardised initial dosage of the trial treatments. In order to reach a consensus, it was decided to use the PRECIS tool to retrospectively determine the pragmatism of the trial’s design.

## Methods

### Applying the PRECIS wheel to BLISTER

The TMG for BLISTER consists of the trial manager, two clinicians with clinical expertise in pemphigoid, a clinician with expertise in trial design and two statisticians. Blank PRECIS wheels, approximately 15 cm in diameter, together with the paper by Thorpe *et al*. [2] containing detailed instructions for completing the wheel were sent to the six TMG members for completion. All members completed their wheels independently apart from the two statisticians, who completed a single wheel together. Some TMG members were unable to complete the wheel using only the guidance given in the paper by Thorpe *et al*. [2] and so extra guidance was provided on scoring each domain in the context of the trial (see Table 1) rather than in the more general context given in their paper. For example, Thorpe *et al*. do not discuss the primary analysis domain in the context of a non-inferiority primary endpoint. It is widely accepted that such an endpoint should be analysed on both intention-to-treat (pragmatic) and per-protocol (explanatory) populations [9], which implies that a non-inferiority trial cannot lie at either extremity of the ‘primary analysis’ continuum. In our study, we scored this domain by considering not only the inclusiveness of the per-protocol definition for the non-inferiority endpoint but also the method of analysis for the co-primary superiority safety outcome. Such co-primary outcomes are often present in non-inferiority studies and must be considered with equal importance.

**Table 1:** Additional guidance for scoring the 10 PRECIS domains in the context of the BLISTER trial

**PRECIS domain**	**Description in the context of BLISTER**
Eligibility criteria	Are most patients who would normally be treated included in the trial? How strict are the criteria?
Follow-up intensity	How similar to normal practice is the patient follow-up?
Primary outcomes	Would those considered to be a treatment success in the trial (for example, three or fewer blisters present at six weeks of follow-up) also be considered a treatment success in practice?
Method of analysing the primary endpoints	Are there many exclusions from the analysis? How inclusive is the definition of per-protocol?
Doxycycline and prednisolone flexibility	Can trial treatments be altered during follow-up or are patients expected to stay on the prescribed dose? Is this any different to normal practice?
Practitioner expertise on doxycycline and prednisolone	Are patients seen by specialists? Would they normally be seen by, for example, general practitioners?
Participant compliance	How closely is this measured? Are there interventions made to maintain or improve compliance?
Practitioner adherence to the protocol	Is the study concerned whether investigators ‘customise’ the trial to suit their own setting? Are all investigators expected to treat patients in the same way?

In addition to this extra guidance, members were also given the option of scoring each domain on a 0 (most explanatory) to 10 (most pragmatic) scale if this was easier. Scores obtained from this method were then mapped onto a wheel by the person collating the results.

Once each wheel had been completed, the distance of the score in each domain from the most extreme explanatory end of the spectrum (the centre) was measured and transformed into a percentage of the total length of a spoke. If the scoring was given on a 0 to 10 scale, percentages were calculated by simply multiplying by 10. The transformed results were then averaged and plotted on a single PRECIS wheel together with the minimum (most explanatory) and maximum (most pragmatic) scores in each domain.

## Results

**Figure 2:**
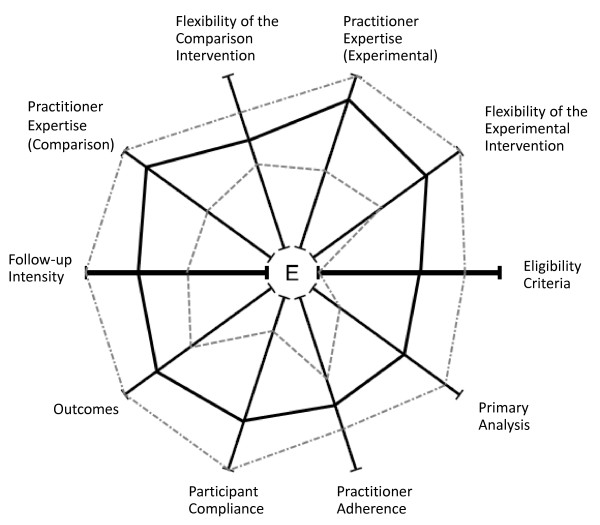
**PRECIS wheel showing the mean of the scores given by BLISTER trial management group members (solid line).** Also presented are the most explanatory scores (inner line) and most pragmatic scores (outer line) given in each domain. Scores were plotted on the wheel using a simple picture editing program.

The scores given in each domain of the PRECIS wheel from each TMG member are shown in Table 2. The overall results, calculated as the mean score in each domain, are shown in Figure 2 along with the most explanatory and most pragmatic scores given by the TMG.

**Table 2:** Summary of scores given by BLISTER trial management group members for each domain of the PRECIS wheel

**Domain**	**Score**					**Mean score (SD)**	**Median score**
	**A**	**B**	**C**	**D**^**a**^	**E**^**a**^		
Eligibility criteria	74	83	0	80	50	57 (35)	74
Flexibility of the experimental intervention	64	86	85	50	100	77 (20)	85
Practitioner expertise (experimental)	45	100	92	100	90	85 (23)	92
Flexibility of the comparison intervention	64	80	80	50	60	67 (13)	64
Practitioner expertise (comparison)	45	98	90	100	100	87 (24)	98
Follow-up intensity	61	89	50	100	70	74 (20)	70
Outcome	79	58	75	100	100	82 (19)	79
Participant compliance	86	84	20	100	80	74 (31)	84
Practitioner adherence	59	77	70	50	80	67 (13)	70
Primary analysis	74	92	20	50	80	63 (29)	74

A to E represent the members of the trial management group. Scores were transformed onto a 0 to 100 scale with 0 representing the most extreme explanatory end of each domain and 100 representing the most extreme pragmatic end. ^a^D and E scored the pragmatism of the trial on each domain out of 10 rather than opting to complete a PRECIS wheel.

As Figure 2 shows, the mean score for each domain is closer to the rim, or the pragmatic end, of the PRECIS wheel than the centre, or explanatory end. Table 2 shows that consensus on the pragmatism of the trial among all TMG members was present in only a few domains. For example, the standard deviation of the scores is relatively low (less than 15) on the ‘practitioner adherence’ and ‘flexibility of the comparison intervention’ domains. In most other domains, the large standard deviations are due to outliers. For example, the ‘participant compliance’, ‘eligibility criteria’ and ‘primary analysis’ arms were considered by one member of the TMG to be almost totally explanatory in contrast to the rest of the group. A number of domains were scored at the pragmatic extremity, suggesting that the trial could be considered entirely true to clinical practice in those domains.

## Discussion

Although we have successfully managed to identify BLISTER as a predominantly pragmatic trial, a number of issues concerning how the PRECIS wheel is completed have become apparent.

Asking only the members of a TMG to independently complete the PRECIS wheel has its advantages and disadvantages. First, independent completion of the wheel ensures results are not influenced by other TMG members. Furthermore, the members of a TMG are expected to be fully familiar with the trial and are arguably in the best position to determine its pragmatism; however, they may have a vested interest in calling the trial pragmatic or explanatory, which could influence their results. To reduce such bias, the members of the data monitoring or trial steering committees, who are independent to the trial but also have enough knowledge on both current clinical practice and the trial itself, could also be asked to complete the wheel and have their results combined and compared with those from the TMG.

One of the main problems encountered during the completion of the wheel for the BLISTER trial was that some TMG members, depending on their expertise, found it difficult to complete certain domains. For example, the statisticians, with limited knowledge of the current clinical practice for treating bullous pemphigoid, found it difficult to complete the practitioner expertise domains. This inevitably led to guesswork but was unlikely to strongly affect the overall result since three of the other TMG members completing the wheel were expert dermatologists and so would provide more accurate scores. By contrast, some of the clinicians found the primary analysis domain tricky since it poses a more statistical than clinical question. Thus it is clear that composition and balance of the team may influence the end result to some extent.

A strength of a PRECIS wheel is that it is able to clearly highlight any inconsistencies or outliers arising through guess work, as described above, or by conflicting viewpoints. There is an argument, therefore, for following up independent scoring of domains with team discussions between those involved in the scoring to resolve misunderstandings and disagreements and to reach a clearer consensus, rather than relying on only one mean score.

In this study we used the PRECIS tool to retrospectively determine the pragmatism of a clinical trial. It should be pointed out that this is not the intended use of the tool, and in certain situations problems can arise from using the tool in this way. For example, if a researcher wishes to retrospectively complete a PRECIS wheel for a particular trial and the only available source of information is the trial report then, despite reporting standards such as the Consolidated Standards of Reporting Trials and its pragmatic trial extension [10], there may be inadequate reporting of some of the PRECIS domains to enable the researcher to complete the wheel fully. Tosh and colleagues suggested that domains with no information available in the trial protocol or elsewhere should be scored at the explanatory extremity [6]. Such an approach may be inappropriate since it biases poorly described trials towards the explanatory end of the spectrum even if in reality they were very pragmatic in their design. In our study, all those who completed a wheel were fully familiar with the trial and its design, and a full trial protocol had already been developed, therefore problems regarding poorly described domains were not of concern.

## Conclusions

Identifying which trials are more pragmatic than others is important since pragmatic trials often have more generalizable results. Classifying a trial as pragmatic could have important implications for funders, policy makers, clinicians, patients and those who conduct systematic reviews and guidelines. The PRECIS wheel is a useful tool for determining the extent of pragmatism of a trial and we recommend that it be more widely used, particularly in the design stage. Furthermore, we suggest that completed wheels are included in the trial protocol or trial report to enable researchers to quickly identify trials with designs more suited to their area of interest.

When using the PRECIS wheel to design a trial, we recommend following the methods used by Riddle *et al*. [3], who showed how use of the PRECIS wheel can help researchers reach a consensus on trial design. In their paper, the team first independently completed separate wheels based on the initial design of their trial and each member’s ideal design. The results and the variability of the scores in each domain were then discussed in detail and a final wheel was then produced by each team member. The variability in the scores given for the final wheel was shown to be lower than that for the initial and ideal wheels showing stronger agreement had been reached.

If the trial has already commenced, we recommend that the PRECIS wheel should also be completed by members of independent committees to the trial as well as members of the trial management group in order to minimise bias.

## Abbreviations

BLISTER, Bullous Pemphigoid Steroids and Tetracyclines Study; PRECIS, Pragmatic-Explanatory Indicator Summary; TMG, Trial management group.

## Competing interests

The authors declare that they have no competing interests.

## Authors’ contributions

DJB drafted the manuscript. HCW conceived the idea for the research and both HCW and AJN helped to draft the manuscript. All authors completed a PRECIS wheel and read and approved the final manuscript.
